# Use of Machine Learning to Predict Individual Postprandial Glycemic Responses to Food Among Individuals With Type 2 Diabetes in India: Protocol for a Prospective Cohort Study

**DOI:** 10.2196/59308

**Published:** 2025-01-23

**Authors:** Niteesh K Choudhry, Shweta Priyadarshini, Jaganath Swamy, Mridul Mehta

**Affiliations:** 1 Department of Medicine Harvard Medical School Boston, MA United States; 2 Decipher Health Delhi India

**Keywords:** diabetes, T2DM, diabetes management, food responsiveness, postprandial glucose response, food intake, diet logs, dietary intake, machine learning

## Abstract

**Background:**

Type 2 diabetes (T2D) is a leading cause of premature morbidity and mortality globally and affects more than 100 million people in the world’s most populous country, India. Nutrition is a critical and evidence-based component of effective blood glucose control and most dietary advice emphasizes carbohydrate and calorie reduction. Emerging global evidence demonstrates marked interindividual differences in postprandial glucose response (PPGR) although no such data exists in India and previous studies have primarily evaluated PPGR variation in individuals without diabetes.

**Objective:**

This prospective cohort study seeks to characterize the PPGR variability among individuals with diabetes living in India and to identify factors associated with these differences.

**Methods:**

Adults with T2D and a hemoglobin A_1c_ of ≥7 are being enrolled from 14 sites around India. Participants wear a continuous glucose monitor, eat a series of standardized meals, and record all free-living foods, activities, and medication use for a 14-day period. The study’s primary outcome is PPGR, calculated as the incremental area under the curve 2 hours after each logged meal.

**Results:**

Data collection commenced in May 2022, and the results will be ready for publication by September 2025. Results from our study will generate data to facilitate the creation of machine learning models to predict individual PPGR responses and to facilitate the prescription of personalized diets for individuals with T2D.

**Conclusions:**

This study will provide the first large scale examination variability in blood glucose responses to food in India and will be among the first to estimate PPGR variability for individuals with T2D in any jurisdiction.

**Trial Registration:**

Clinical Trials Registry-India CTRI/2022/02/040619; https://tinyurl.com/mrywf6bf

**International Registered Report Identifier (IRRID):**

DERR1-10.2196/59308

## Introduction

Type 2 diabetes mellitus (T2D) is the leading cause of chronic kidney disease, end-stage renal disease, blindness, and nontraumatic amputation; it also substantially increases the risk of myocardial infarction, stroke, and heart failure [1]. Its prevalence is particularly high in India, which is now the most populous country in the world. As of 2023, more than 100 million people living in India have diabetes, representing more than 11% of the population and an additional 136 million have prediabetes [2]. These numbers are anticipated to continue to grow rapidly. The lifetime risk of T2D among 20-year-olds who are obese and living in India is estimated to be more than 86% [3]. The rising prevalence of this condition in India is believed to be the result of changing diets, increasingly sedentary occupations, lower levels of physical activity in the context of urbanization, and rapidly increasing rates of obesity [4].

These trends are particularly concerning because of important differences between the presentation and consequences in T2D among individuals of South Asian origin compared with other racial and ethnic groups. These differences, which often referred to as the “Indian Phenotype” or “South Asian Phenotype” [5-7], are characterized by the onset of T2D at a younger age and substantially lower BMI than people of other races and ethnicities [8-10]. Individuals of Indian origin have higher levels of insulin resistance (and for longer periods of time) and premature beta-cell failure [7]. They are more likely to develop the fatal complications of T2D, most notably heart disease [7]. These features are thought to result from a mix of lifestyle, epigenetics, and fetal programming factors [7,11,12].

The fundamental goal of diabetes management is to maintain near-normal glucose levels. A variety of self-management behaviors, in particular adherence to diet and regular exercise, are central to this goal. An extensive body of evidence demonstrates that aiding patients with T2D with self-management behaviors is associated with improvements in a wide range of outcomes including knowledge, self-care behaviors, weight, quality of life, hemoglobin A_1c_ (HbA_1c_), all-cause mortality, and health care costs [13,14].

Guidelines recommend that nutritional guidance be personalized based on nutritional status, lifestyle, and metabolic goals [15]. Despite this, most dietary advice for individuals with T2D remains generic emphasizing reductions in calories and minimization of carbohydrates [16]. However, there are marked interindividual responses in postprandial glucose response (PPGR) [17]. A study conducted in Israel found substantial PPGR variability to standardized meals for individuals without diabetes [18]. Similar data has been generated in the United Kingdom, the United States, and China [19-21].

There have been no studies characterizing food responsiveness among individuals living in India and virtually no published data, from any judication, in the variability in PPGR for individuals with T2D [22]. Given the unique Indian diabetes phenotype and differences between Indian and western diets, in specific much higher rates of carbohydrate consumption overall [23] and the centrality of white rice and refined wheat [24], there are very likely to be differences in blood glucose responses to food and exercise in India than observed elsewhere, just as there have been in Indians’ responses to diabetes medications [25]. Accordingly, the goal of this study is to characterize and identify factors associated the variability in PPGR among individuals with T2D in India.

## Methods

**Figure 1:**
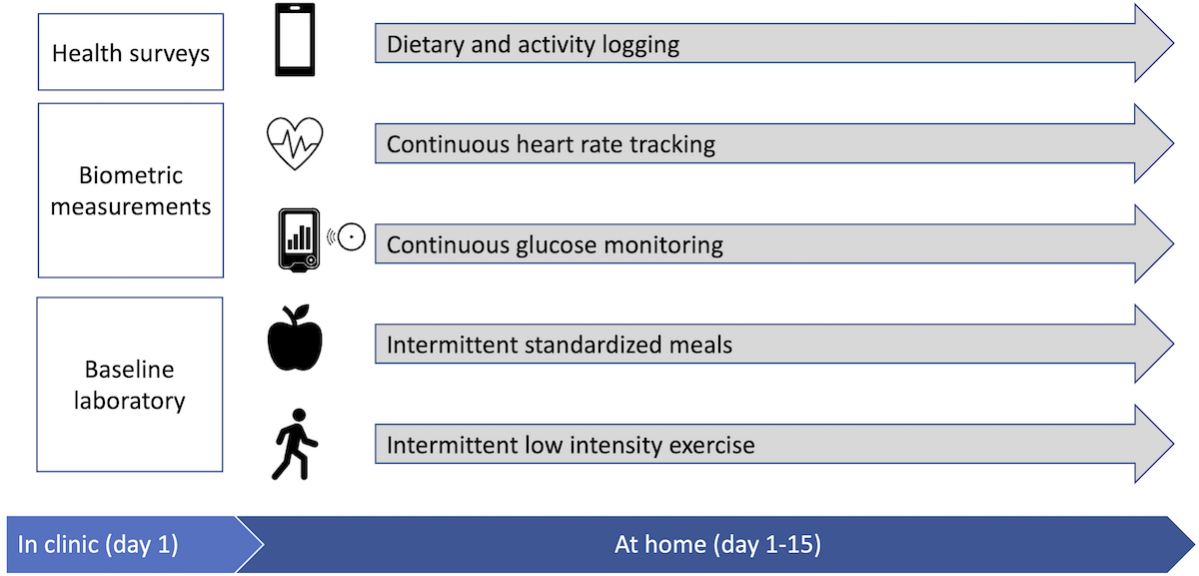
Overall study design.

This prospective cohort study seeks to evaluate the relationship between PPGR and self-management activities including diet, exercise, and other daily routines, for individuals with T2D in India (Figure 1).

### Study Setting

This trial is being conducted at 14 outpatient clinics in geographically distinct regions across India with population sizes ranging from 1.2 to 34 million. Sites were identified and managed by IQVIA, a multinational contract research organization, and were included if they specialized in the care of individuals with diabetes, had an established research infrastructure for the conduct of diabetes-related studies including a site principal investigator who is a diabetologist (with clinical training in endocrinology or internal medicine), a local ethics committee to provide study oversight, and a sufficient volume of potentially eligible patients. Study enrollment began in May 2022.

### Eligible Participants and Enrollment

The study population consists of adults with diabetes and suboptimal disease control, classified as HbA_1c_ ≥7. Complete inclusion and exclusion criteria are summarized in Textbox 1.

**Eligibility criteria required for participants to be enrolled in the study.**

**Inclusion criteria**

- Age ≥ 18 and <75 years.
- Physician-diagnosed type 2 diabetes treated with ≥1 oral hypoglycemic agents
- Hemoglobin A_1c_≥ 7.0% recorded within the past 30 days.
- Mobile phone capable of running protocol-specified apps.
- Functional English literacy.

**Exclusion criteria**

- Unable or unwilling to provide informed consent or comply with the study-specified procedures.
- Current use of prandial insulin including a continuous insulin infusion pump.
- Currently pregnant or planning to become pregnant.
- Estimated life expectancy of ≤12 months.
- Active cancer.
- Myocardial infarction or stroke in the last 6 months.
- Receiving or planned to initiate dialysis for end-stage renal disease.
- Receiving oral or intravenous steroids.
- Any contraindication to using a continuous glucose monitor.

Potentially eligible patients are identified from clinic records and are invited to attend an in-person screening visit at which time eligibility is confirmed and written informed consent obtained. Consenting patients are asked to provide sociodemographic and medical information (specifically, age, sex, predominant diet, health conditions, family history, and current medications) and to complete baseline surveys including the World Health Organization’s STEPwise Approach to Non-Communicable Disease Risk Factor Surveillance (STEPS) survey [26], World Health Organization-Five Well-Being Index (WHO-5) [27]. Diabetes Distress Scale [28], Wilson Adherence Scale [29], and the Pittsburgh Sleep Quality Index [30].

Biometric data including blood pressure, heart rate, weight, height, and body measurements at the upper arm, thigh, calf, waist, and hips, are collected by study coordinators at each site. Finally, enrolled participants provide blood samples including a complete blood count, HbA_1c_, blood electrolytes, creatinine, cholesterol, as well as urinalysis.

After completing baseline assessments, participants are fitted with an Abbott Freestyle Libre continuous glucose monitors (CGM) sensor on their upper, nondominant arm and are provided with a Xiaomi Mi Band Smart Wristband (heart rate monitor) and a Roche Accu-Chek glucometer with testing supplies, and dietary supplements to be consumed with their standardized meals (refer to Follow-up Procedures section). Study-specific apps are downloaded on to the participants’ smartphones to allow them to log dietary intake and synchronize their continuous glucose and heart rate monitors. As a back-up, participants are given a paper dietary logbook and a Freestyle Libre CGM reader with which to collect protocol-specified data.

### Follow-Up Procedures

Participants are followed for 14 days. They are instructed to wear the CGM and heart rate monitor. The heart rate monitor is to be always worn, including during sleep, and only removed for recharging. Participants are also asked to check their capillary glucose on days 2 through 6 before breakfast and dinner.

Participants log their full dietary intake using the study app or logbook over the 14-day study period, including all standardized test meals and free-living foods (including snacks), beverages (including water), and medications. Participants also log all exercise.

Participants are required to consume protocol-specified meals and to perform light activity, as described in Table 1. The standardized meals consist of vegetarian breakfast foods which participants are to prepare in their homes.

The meals vary in their proportion of carbohydrate, fiber, protein, and fat (refer to Table 2).

**Table 1:** Meal schedule during the at-home study period.

Day and timing			Meal type
**1**			
	Breakfast	Fasting	
	Lunch	As desired	
	Dinner	As desired	
**2**			
	Breakfast	Perceived healthy meal	
	Lunch	Post meal exercise	
	Dinner	Typical mixed protein with dinner	
**3**			
	Breakfast	Fiber supplement with standardized test meal	
	Lunch	Premeal exercise	
	Dinner	Alternative mixed protein with dinner	
**4**			
	Breakfast	Fiber supplement before standardized test meal	
	Lunch	As desired	
	Dinner	As desired	
**5**			
	Breakfast	Protein supplement with standardized test meal	
	Lunch	As desired	
	Dinner	Alternative mixed protein with dinner	
**6**			
	Breakfast	Protein supplement before standardized test meal	
	Lunch	Added fiber with lunch	
	Dinner	Alternative mixed protein with added protein	
**7**			
	Breakfast	Regular breakfast	
	Lunch	Added fruit	
	Dinner	Cooled carbohydrate	
**8**			
	Breakfast	Regular breakfast with added protein	
	Lunch	Added fruit with protein	
	Dinner	Regular carbohydrate	
**9**			
	Breakfast	Protein followed by carbohydrate	
	Lunch	Water 30 minutes before lunch	
	Dinner	As desired	
**10**			
	Breakfast	Standardized test meal	
	Lunch	As desired including dessert	
	Dinner	Protein followed by carbohydrates and vegetables	
**11**			
	Breakfast	Standardized test meal with postmeal exercise	
	Lunch	As desired including desert and cinnamon	
	Dinner	Vegetables followed by protein then carbohydrates	
**12**			
	Breakfast	Standardized test meal with postmeal exercise	
	Lunch	As desired	
	Dinner	As desired finishing eating by 8 PM	
**13**			
	Breakfast	Water before late breakfast + postmeal exercise	
	Lunch	Low glycemic index lunch	
	Dinner	Low glycemic index dinner + finish dinner by 8 PM + exercise after meal	
**14**			
	Breakfast	Water before late breakfast + exercise after meal	
	Lunch	Low glycemic index lunch + exercise after meal	
	Dinner	Low glycemic index dinner + finish dinner by 8 PM + exercise after meal	

**Table 2:** Nutritional composition of standardized test meals.

Meal characteristics	Meal type		
	Carbohydrate	Added fiber	Added protein
Example meal	2 aloo parathas with curd	2 aloo parathas with curd + fiber supplement	2 aloo parathas with curd + protein supplement
Energy (kcal)	474.4	506.4	594.4
Carbohydrate (g)	61.5	64.1	62.9
Fat (g)	12.5	12.5	14.6
Protein (g)	7.7	7.8	31.9
Fiber (g)	8.3	21.9	8.3
Carbohydrate (% energy)	51.9	50.6	42.3
Fat (% energy)	23.8	22.3	22.1
Protein (% energy)	6.46	6.05	21.4

Participants are instructed to fast for a minimum of 8 hours before and 3 hours after consuming the standardized breakfast meal; during these fasting periods, limit exercise and drink only still (not sparkling) water, tea, or coffee in moderation; and eat the meal, in its entirety, within 20 minutes. After completing the postmeal fasting period on standardized test meal days, participants may consume other foods as they normally would unless there are other meal modifications specified by the protocol later the same day.

On other days, participants are asked to consume normal foods with protocol-specified constraints. For example, on different days, participants vary the types of mixed protein (eg, different types of lentils with or without added protein), ordering with the type of foods consumed (eg, protein before carbohydrate vs protein with carbohydrate), consume water before their meal, go for a walk after eating, or eat what they perceive to be a healthy meal. Where applicable, participants are given several options as to which of their usual foods are acceptable for each protocol specified food modification.

During the follow-up period, participants are contacted by phone and text messages to ensure protocol compliance. For participants using the study-specific smartphone apps, a web dashboard is used to monitor the completeness of dietary logging and CGM scanning, with outreach to participants initiated when missing data is detected. Participants using paper logs and a CGM reader, and therefore for whom no dashboard information is available, are contacted daily to ask about protocol compliance. Ad hoc midstudy visits are used to further ensure accurate data collection. If the outreach identifies a problem with a CGM (ie, it was damaged, fell off, or malfunctioned), study staff provide a replacement within 24 hours during which time participants are asked to pause their meal protocol and restart once their CGM has been reapplied and recalibrated. If a test meal was not consumed as intended, participants are provided with the option to repeat the meal.

On day 15, participants are asked to remove their CGM. If need be, study staff help record or correct missing or inaccurate food, activity, and medication data.

### Statistical Analysis Plan

The study’s primary outcome is PPGR. Following the Wolever and Jenkins method [31], as adapted by Zeevi et al [18], Mendes-Soares et al [20], and Berry et al [32], logged meal times and continuous glucose measurements will be used to calculate the incremental area under the curve. Before conducting analyses, meals logged less than 30 minutes apart will be merged and meals logged within 90 minutes of other meals will be removed. Meals that are very small (<15 g and <70 calories), very large (>1 kg), with implausibly low PPGR values (ie, a PPGR < 5 mg/dL·h after consuming ≥ 40 g of carbohydrates), that are incompletely logged, and which are consumed in the first and last 12 hours of the CGM connection will also be removed. To reduce noise, the median of all glucose values from the 30-minute period before the meal will be taken as the initial glucose level, above which the incremental area will be calculated. Meals that had incomplete glucose measurements in the time window of 30 minutes before and 2 hours after the logged mealtime will be filtered out.

Descriptive statistics will be used to plot the range of PPGRs responses to standardized test meals as well as the correlation between PPGR and the nutritional composition of the logged meals (ie, carbohydrates, fat, and protein).

A machine learning predictor will be developed based on stochastic gradient boosting regression (XGBoost, version 2.2.1; The XG Boost Contributors) [33] using the XGBRegressor class. PPGR will be predicted as the sum of predictions from thousands of decision trees. Trees will be inferred sequentially, with each trained on the residual of all previous trees. The features incorporated in each tree are selected by an inference procedure from features representing meal content (ie, calorie, protein, carbohydrate, and fiber content), meal timing, baseline demographics (ie, age, sex, predominant diet, health conditions, family history, and current medications), baseline survey results (ie, WHO STEPS, WHO-5, Diabetes Distress School, Wilson Adherence Scale, and Pittsburgh Sleep Quality), baseline biometric values (ie, blood pressure, heart rate, weight, height, and body measurements at the upper arm, thigh, calf, waist, and hips), baseline laboratory values (complete blood count, HbA_1c_, blood electrolytes, creatinine, cholesterol, and urinalysis), as well as CGM, heart rate, and activity data.

Performance will be assessed by holding out 30% of the sample and using 5-fold cross-validation in which cross-validation participants are divided into 5 groups, the model will be trained on the other 4 parts. Random datasets of the same size as the original will be sampled with replacement from the original dataset, and the entire training and validation process will be repeated. The performance will be measured by the ability to accurately predict meals reported by the held-out participants. Prediction results will be aggregated, and Pearson product moment correlation with the measured PPGRs will be reported. The SE for the calculated performance will be assessed using at least a thousand iterations of bootstrapping until the errors stabilize. Model discrimination will be assessed using a binary cut-point for PPGR, set at the 50th percentile of all observed PPGR values, plotting a receiver operating characteristic curve (ROC) and then calculating the area under the ROC.

### Sample Size Considerations

A total of 1050 individuals will be targeted for recruitment. Assuming a 5% loss to follow-up, this corresponds to 1000 evaluable individuals at the end of the study. The study has been designed to predict postprandial glucose responses based on individual characteristics and 1000 participants followed for 14-days will result more than 4 million glucose readings (assuming glucose readings from the CGMs every 5 minutes) and 42,000 meals (assuming 3 meals per participant per day). This volume of data will also provide more than 80% power to detect correlations of a magnitude of *r*=0.13 (*R*^2^=0.02) with *P*<.005. We will also be sufficiently powered to detect effects of *r*=0.17 (*R*^2^=0.03, ie, explaining 2.7% of interindividual variation) with *P*<.001, that is, accounting for 5000 independent hypothesis tests.

### Ethical Considerations

This study was approved by the following ethics committees at all institutions enrolling patients: SShrey Hospital Institutional Ethics Committee (DHINDIA_2021_01A04), Inamdar Multispecialty Hospital Ethics Committee (DHINDIA_2021_01A04), Neelima Hospitals Institutional Ethics Committee (DHINDIA_2021_01A04), Jaipur National University Institutional Ethics Committee (JNUIMSRC/IEC/2022/06), Maharaja Agrasen Hospital Institutional Ethics Committee EP/F-174), Ganesh Shankar Vidyarthi Memorial Medical College Ethics Committee (EC/72/March/2022), Mar Augustine Golden Jubilee Hospital Institutional Ethics Committee (DHINDIA_2021_01A04), Medisys Clinisearch Ethical Review Board, Dayanand Medical College and Hospital Drug Trials Ethics Committee (DMCH/DTEC/2020/1242), Chennai Meenakshi Multispecialty Hospital Ethics Committee (CMMHEC/22/02), Sparsh Hospital Institutional Ethics Committee (ZZA78309), Medical College of Kolkata Institutional Ethics Committee (MC/KOL/IEC/SPON/1296/03/22), Ethics Committee Downtown Hospital, and CHL-Hospitals Integrity Ethics Committee.

## Results

Data collection commenced in May 2022, and the results will be ready for publication by October 2025. Results from our study will generate data to facilitate the creation of machine learning models to predict individual PPGR responses and to facilitate the prescription of personalized diets for individuals with T2D.

## Discussion

### Study Implications

This study will provide the first large scale examination of variability in blood glucose responses to food in India and will be among the first to estimate PPGR variability for individuals with T2D in any jurisdiction. We hypothesize that there will be substantial interindividual variability in PPGR and that, based on the data collected from this study, machine learning models will be able to accurately predict individual PPGR responses. This will facilitate the prescription of truly personalized diets for individuals with T2D.

These results will be particularly important in the context of the rapidly rising prevalence of T2D in India [34]. Along with medications and physical activity, diet is a key tenant of effective blood glucose control [15]. Like in other jurisdictions, guidelines call for individualization of meal planning, which is sometimes referred to as “Medical Nutritional Therapy.” Despite this, personalization of dietary plans are generally based upon broad constructs like age, activity level, health status, and preferences, and, for all patients, tend to emphasize overall calorie reductions and minimization of carbohydrate [16]. especially added sugars and refined grains, in favor of the consumption of nonstarchy vegetables and foods that are high in protein [35].

However, emerging data demonstrates that there are marked interindividual responses to food [17], attributed to differences in physical activity [36], gut microbiome [18,19,37], and genetics [38], including in variations in skeletal glucose transporters related to insulin resistance [39]. For example, a study conducted in the United States among nondiabetic individuals with a mean BMI of 27 found PPGR to a standardized meal of bagel and cream cheese ranged from 6 to 94 mg/dL·h [20]. A similar study conducted in Israel enrolled nondiabetic individuals of whom 3-quarters had a BMI ≥ 25 and found mean PPGR to bread and butter of 44 mg/dL·h but the bottom decile had responses of ≤ 15 mg/dL·h and the top decile has responses ≥ 79 mg/dL·h [18]. Similar data have been generated for individual without diabetes in the United Kingdom and the United States [19,20], and for individuals with type 1 diabetes in Israel [40].

There is exceptionally limited data for variability in PPGR for individuals with T2D in the peer-reviewed although it is highly likely that such variability exists [22]. The primary goal of our study is to fill this void and to generate an India-specific machine-learning models on the basis of which PPGR can be predicted with high accuracy for T2D. Similar models have been built in other jurisdictions. For example, a machine learning algorithm trained on CGM data, dietary, activity, anthropometrics, and gut microbiota for nondiabetic individuals in Israel was much more accurate at predicting PPGR than generic models based on the carbohydrate content or the amount of calories in a meal [18]. A separate US-based study had similar findings [20].

Among individuals with diabetes, a study in the Netherlands that included a small number of individuals with T2D along with individuals with prediabetes and normal glucose metabolism, a machine learning model based on CGM data was highly accurate in predicting future glucose values but this study did not specifically evaluate the ability to predict PPGR [41]. A US study of 1000 patients of whom 1-quarter had T2D found that a machine learning model trained on CGM, HRM data and food logs was highly accurate at predicting PPGR but this study has, to our knowledge, only been published in abstract form [22]. These studies have all relied on CGM data to make their predictions. While these devices are increasingly used, practice guidelines do not recommend their long-term use for most individuals with T2D [42]. Accordingly, a key goal of our study will be to explore the ability to predict PPGR response without reliance on CGM data or with very limited blood glucose data from patients.

### Limitations

There are several limitations to our approach. Our approach is purposely pragmatic and is intended to simulate real-world circumstances for individuals with T2D living in India. Similar to studies conducted in other jurisdictions, we rely on self-reported dietary and activity information. And, while we are auditing patient logs on an ongoing basis, there may nevertheless be issues with protocol adherence that may undermine the accuracy of the data we collect. Participants are being recruited from clinics, predominantly caring for individuals with diabetes, are required to have functional English literacy and a cellphone capable of running study specific devices. Thus, our results may not be fully generalizable to patients who do not fulfill these criteria. Finally, some of the enrollment has overlapped with the COVID-19 pandemic, which may have influenced access to health care, dietary practices, and glucose control for individuals with T2D.

### Conclusion

In conclusion, this study will provide the first large scale examination variability in blood glucose responses to food in India and will be among the first to estimate PPGR variability for individuals with T2D in any jurisdiction. Results from our study will generate data to facilitate the creation of machine learning models to predict individual PPGR responses and to ultimately facilitate the prescription of truly personalized diets for individuals with T2D.

## Abbreviations

CGM: continuous glucose monitor
HbA_1c_: hemoglobin A1c
PPGR: postprandial glucose response
ROC: receiver operating characteristic
STEPS: STEPwise Approach to Non-Communicable Disease Risk Factor Surveillance
T2D: type 2 diabetes mellitus
WHO-5: World Health Organization-Five Well-Being Index

## Data Availability

The datasets generated during and analyzed during this study are not publicly available as they will be used for commercial application. Data may be available on reasonable request after executing appropriate data use and licensing agreements.
